# Innovative Delivery and Release Systems for Antioxidants and Other Active Substances in the Treatment of Cancer

**DOI:** 10.3390/ph16071038

**Published:** 2023-07-21

**Authors:** Zerrin Sezgin-Bayindir, Sonia Losada-Barreiro, Sofía Fernández-Bravo, Carlos Bravo-Díaz

**Affiliations:** 1 Department of Pharmaceutical Technology, Faculty of Pharmacy, Ankara University, Ankara 06560, Turkey; zerrin.sezgin@pharmacy.ankara.edu.tr; 2 Departamento de Química-Física, Facultade de Química, Universidade de Vigo, 36200 Vigo, Spain; cbravo@uvigo.es; 3 Odontology Department, Primary Health Care Unit, Galician Health Service (SERGAS), Camiño do Lodairo s/n, 15570 Narón, Spain; sofiafbravo@gmail.com

**Keywords:** antioxidants, delivery systems, anticancer, controlled release, bioavailability

## Abstract

Cancer is one of the major diseases leading to death worldwide, and the fight against the disease is still challenging. Cancer diseases are usually associated with increased oxidative stress and the accumulation of reactive oxygen and nitrogen species as a result of metabolic alterations or signaling aberrations. While numerous antioxidants exhibit potential therapeutic properties, their clinical efficiency against cancer is limited and even unproven. Conventional anticancer antioxidants and drugs have, among others, the great disadvantage of low bioavailability, poor targeting efficiency, and serious side effects, constraining their use in the fight against diseases. Here, we review the rationale for and recent advances in potential delivery systems that could eventually be employed in clinical research on antioxidant therapy in cancer. We also review some of the various strategies aimed at enhancing the solubility of poorly water-soluble active drugs, including engineered delivery systems such as lipid-based, polymeric, and inorganic formulations. The use of cyclodextrins, micro- and nanoemulsions, and thermosensitive smart liposomes as useful systems for the delivery and release of poorly aqueous-soluble drugs, improving their bioactivity and stability, is also addressed. We also provide some details on their formulation processes and their use in a variety of medical applications. Finally, we briefly cover a case study specifically focused on the use of delivery systems to minimize oral cancer and associated dental problems.

## 1. Introduction

**Figure 1:**
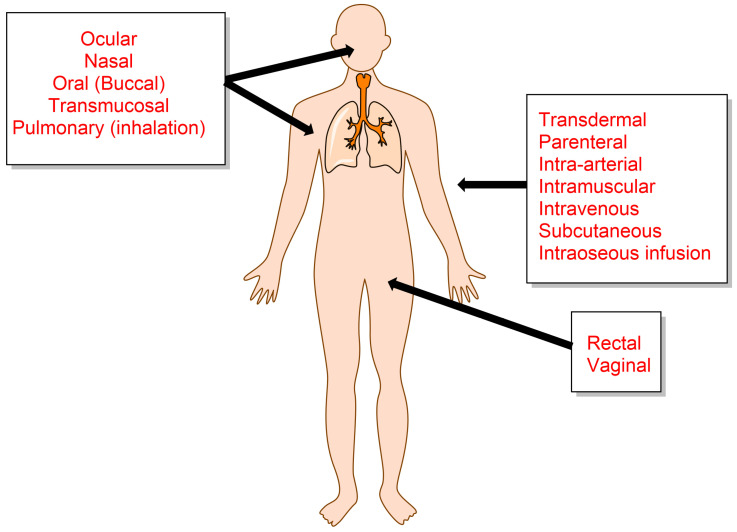
Selected conventional anatomical channels for systemic drug delivery.

Cancer has been, and still is, one of the major diseases leading to death and, consequently, has been the focus of enormous scientific research and study. Despite the thousands of reports on the topic, the fight against cancer is still challenging because its origin and subsequent development are still far from being completely understood, making it a major lethal disease for people. Historically, humans have treated symptoms and diseases with natural remedies extracted from roots, leaves, seeds, berries, flowers, etc. For example, ancient Chinese and Egyptians employed plants and herbal therapies for medical purposes [1,2,3]. Later, in the early 19th century, natural medicine evolved as new methods to identify and synthesize plant compounds that were administered in higher concentrations, to patients [3]. Several routes are commonly employed to deliver drugs of interest [4,5] to the human body; some of the most common routes are illustrated in Figure 1. The choice of one or another route basically depends on the type of disease, the product available, and the desired effect [6].

Cancer is usually associated with increased oxidative stress, characterized by the overproduction of reactive oxygen species (ROS) as a result of metabolic alterations or signaling aberrations. Such ROS cannot be quenched by endogenous antioxidants (AOs), leading to alterations of the redox homeostasis equilibrium that are essential for biological functions and, finally, pathology. Usually, ROS levels are tightly regulated by the antioxidant enzymatic and non-enzymatic systems, and cells respond to an eventual overproduction of ROS by modifying metabolic and genetic reprogramming, increasing the production of NADPH, superoxide dismutases (SODS), glutathione, and thioredoxins in an attempt to return to homeostatic levels [7,8,9].

When the ROS levels exceed the non-toxic amounts, ROS can cause oxidative damage to nucleic acids, proteins, and lipids, resulting in fragmentation of enzymes and structural proteins, membrane damage, and eventually gene mutations and even pro-oncologic signaling activation [10,11].

Because oxidative stress plays an important role in carcinogenic processes and in cancer progression, researchers explored the use of antioxidants for the treatment of cancer [12]. Among them, the Nuclear factor erythoid 2-related factor 2 (NRF2), vitamins, *N*-acetylcisteine, NADPH oxidase, and numerous polyphenols have been explored in the last few decades [13]. Some of them have potential as anticancer drugs, and multiple therapeutic strategies have been explored in pre-clinical and clinical research studies. Their use, however, requires the development of appropriate delivery systems to transfer the active antioxidants to target sites without difficulty during and after delivery [13,14]. Specifically, increasing the solubility and bioavailability of active antioxidants or other drugs is crucial in the development of biocompatible pharmaceutical formulations for therapeutical cancer, and the development of such systems has become an active research area [7,13,15].

Here we will review common delivery systems that could eventually be employed to deliver antioxidants and other anticancer drugs to specific target sites. Techniques of preparation such as nanofabrication (extrusion, high-pressure homogenization, etc.), three-dimensional printing, and electrospinning are not covered because excellent reviews on the topic are available in the literature [16,17,18,19]. For the sake of simplicity and clarity, hereafter we will employ the terms “antioxidant” and “drug” to design those molecules, endogenous or exogenous, that have some antioxidant capability and that, eventually, may also have other therapeutic uses.

An important factor that is also addressed in the present work (and in drug delivery) is related to the time required for a drug to be released into the body [20,21]. The release time can be modified by controlling different properties of the delivery system, designing them for immediate, non-immediate, site-specific, or sustained release, so that the performance—effectiveness, patient compliance, and safety—of specific drugs can be increased. Another important factor in medication delivery is the amount of time it takes for a drug to be released into the body. Drug delivery can be modified and controlled by different properties, so researchers exploit several methods for the transfer of drugs [21,22].

## 2. Reactive Oxygen Species in Cancer

**Figure 2:**
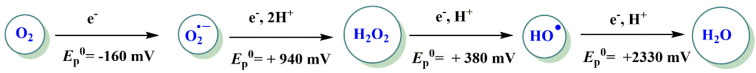
Formation of ROS through electron-transfer reactions. The redox states of oxygen species and their standard redox potentials are shown. The standard concentration of oxygen was 1 M [26].

Reactive oxygen species are a group of highly reactive oxidative molecules formed as normal byproducts of numerous cellular processes that are associated with cancer and other diseases because their basal levels are higher than those typically found in normal cells [23]. Molecular oxygen is a poor univalent electron acceptor and consequently a poor oxidant, but under certain conditions it can evolve to form ROS. Figure 2 shows the sequential steps involved in the formation of different oxygen-centered radicals and oxygen-centered nonradicals from molecular oxygen [24,25].

**Figure 3:**
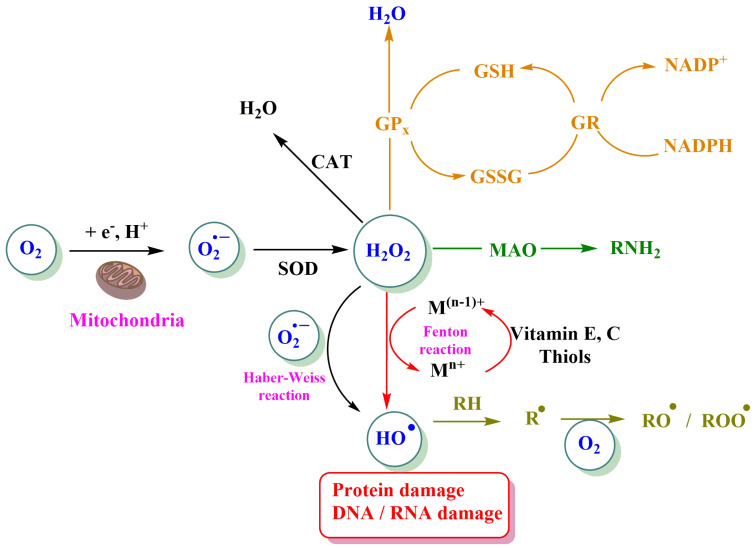
Different pathways involved in the formation and loss of ROS. The generation of superoxide (O_2_^∙−^) from molecular oxygen is mediated either by the NADPH oxidase complex or, in mitochondria, by cytochrome c peroxidase or xanthine oxidase. GPx: Glutathione peroxidase, GR: glutathione reductase, GSH: reduced glutathione, GSSG: glutathione disulfide or oxidized glutathione, SOD: superoxide dismutase, CAT: catalase, MAO: monoamine oxidase, and RH: lipid. Reprinted/adapted with permission from Ref. [14].

ROS are generated by cells as a response to intracellular signaling and extracellular stimuli in the mitochondrial electron transport cell, endoplasmatic reticulum, and peroxisomes (Figure 3). The production of mitochondrial ROS is associated with the metabolism of glucose, fatty acids, and amino acids. ROS can be eliminated by various enzymatic antioxidant systems, including SODs, GSH peroxidases, peroxiredoxins, and catalases. Additionally, ROS can be eliminated by non-enzymatic antioxidant systems, including GSH (a tripeptide composed of glutamate, cysteine, and glycine) and TRXs (thioredoxin reductase and NADPH) [7,23].

The formation of ROS through these electron transfer processes can be anticipated based on the one-electron redox potentials in water (Figure 2) and Table 1. For the sake of comparisons, Table 2 also includes the one-electron redox potentials of various other ROS of biological importance, as well as the one-electron redox potentials of some ROS scavengers [25,27].

When, for any reason, there is an imbalance between the production of oxidants and their quenching by antioxidants, high levels of ROS may be present, and when they are in high concentrations, ROS cause damage to proteins, nucleic acids, lipids, membranes, and other organelles, leading to deterioration of the cell and eventually to cell death. However, ROS is necessary for signaling, acting as signal transducers that activate various vital cell functions, including proliferation, migration, invasion, and angiogenesis. Thus, low to moderate levels are necessary, and the delicate balance between their production and inactivation by antioxidants needs to be controlled. To control the intracellular levels of ROS (i.e., the cellular redox homeostasis), cancer cells defend themselves from high concentrations of ROS and respond to oxidative stress by inducing the transcription of antioxidant enzymes; e.g., the transcription factor NRF2 is a pivotal regulator of antioxidant responses that is activated and overexpressed in cancer to promote cell survival [13].

Given the important role of ROS in cancer, it follows that modulating ROS levels is a promising cancer strategy. Antioxidants can reduce cancer incidence and progression, but the factors that limit the anticancer activity of antioxidants are not fully understood. These factors include the use of pharmacological but not dietary doses (based on in vitro studies) whose activity can be modified by the complex in vivo conditions, and, most importantly, antioxidants may distribute unevenly in different tissues and may not function because of their low bioavailability and/or bioaccesibility in specific organs. Exogenous antioxidants are usually provided through diet since their synthetic pathways are usually present in microbial and plant cells. Vitamin C (ascorbic acid), vitamin E (α-tocopherol), carotenoids, polyphenols, and curcumin are some of the current antioxidants commonly employed in the fight against cancer. Consumption of antioxidant-rich superfoods does not compensate for unhealthy habits and may even be dangerous because a certain level of ROS is needed for signaling and cell function. Antioxidant (and other drug) supplements may have different health benefits, including synergistic effects with other substances present in cells. Natural antioxidants are well-known for their capacity to contribute to reestablishing cell homeostasis under oxidative stress. However, the efficiency of these compounds is usually studied individually, and their synergistic effects are lacking. Several studies have addressed the intracellular generation of ROS by human monocytes (TPH-1) cells [29], a well-known model for studying the effects of toxins and/or the development of new scavengers. Ben Mrid et al. [29] employed TPH-1 cells as a model to evaluate the cytoprotective effect of four natural compounds and their mixtures on H_2_O_2_-induced oxidative stress, analyzing their potential molecular mechanisms to avoid H_2_O_2_-oxidative damage. Results showed that the addition of natural compounds, especially the synergistic effect of curcumin and rosmarinic acid, could act as cytoprotective and immunostimulant agents against H_2_O_2_-induced.

On the other hand, antioxidants certainly need to be targeted through an appropriate delivery system. Indeed, an improvement in the delivery systems of antioxidants should facilitate the development of novel therapeutic agents that might be much more effective in the prevention and treatment of cancer [23,30].

Antioxidant (or drug) delivery systems (ADS or DDS) are designed to improve the pharmacological and therapeutic properties of drugs administered by different routes. Many of the early problems that hindered the clinical applications of particulate ADS have been overcome, with several ADS formulations of anticancer and antifungal drugs now approved for clinical use. Furthermore, there is considerable interest in exploiting the advantages of DDS for in vivo delivery of new drugs derived from proteomics or genomics research and for their use in ligand-targeted therapeutics. In the next sections, we will provide an overview of these systems, their importance, and their uses.

## 3. Antioxidant-Loaded Delivery Systems as Emerging Therapeutic Strategies against Cancer Diseases

Research on bio-based drug delivery systems has gained substantial attention in the last few years, and various research lines aimed at developing formulations to target specific sites in cancer therapy are currently being explored [6]. Multifunctional drug delivery following biomaterials, especially natural polymers that can be combined in therapeutic approaches to oral diseases, has a promising perspective. Renewable polyphenols, such as lignin, may be applied for the encapsulation and controlled release of low-stable natural substances [31]. Examples of the encapsulation of bioactive natural substances inside lignin nanoparticles were reported, including the encapsulation of resveratrol, curcumin, and ascorbic acid esters such as 3-O-ethyl-L-ascorbyl-6-ferulate and 3-O-ethyl-L-ascorbyl-6-palmitate [31]. New drug delivery systems need to be developed for better delivery and controlled release, requiring the interdisciplinary collaboration of chemists, biologists, pharmaceuticals, engineers, and doctors requiring constant rethinking and redesigning of formulations to include new bio-active products [6].

The antioxidant properties of natural polyphenols have attracted the attention of the scientific community as promising anticancer agents. However, their poor bioavailability, high dosage requirements, and rapid clearance make effective administration methods necessary to ensure their absorption, bioavailability, and promissory beneficial effects.

Different types of biodegradable delivery systems, such as organic, metal, polymeric nanoparticles, and lipid-based formulations, are currently being evaluated for specific therapeutic agents. Delivery systems, designed at nanoscale sizes and employing biocompatible materials, have emerged as a strategy for targeted drug delivery as well as a diagnostic tool. The aim of such delivery systems is manifold and includes: (1) to minimize the frequency of cancer patient dosages and any side effects; (2) to control the release of the drug at a pre-established, constant rate for as long as possible; (3) to provide high efficiency, low toxicity, and enhance patient compliance; (4) to offer personalized treatment for patients by employing individual information such as genetic, historical factors, and biomarker data [32]; and (5) the specific conditions of the environment where the action of the antioxidant is required [32]. For example, there is a substantial gap between epithelial cells in the blood vessels in cancerous tissues, leading to an imperfect vascular architecture and low lymphatic drainage. Tailored delivery systems can cross these gaps and eventually accumulate in tumor tissue, improving the enhanced permeability and retention (EPR) effect (passive transport) [33].

**Figure 4:**
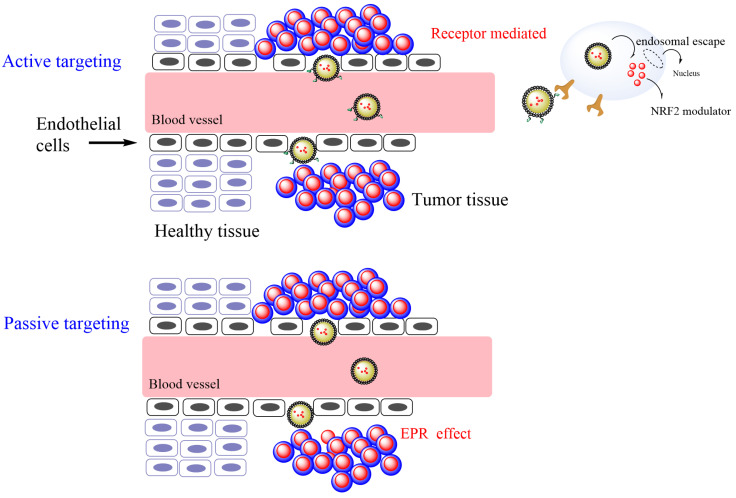
Enhanced permeability of a therapeutic antioxidant (NRF2 modulator) through active and passive targeting of tumor cells by employing nanoemulsions as a delivery system.

The development of potential drug delivery systems may also be tailored to consider the significant differences that exist between the growth of normal tissues and tumors on the basis of different aspects, including angiogenesis, oxygenation, perfusion, and vascular abnormalities [34]. Several studies have reported on the use of nanoemulsion-based carrier systems for passively targeting cancer (Figure 4) [34]. TOCOSOl^®^ is a representative nanoemulsion employed to improve the accumulation and efficiency of a chemotherapeutic antioxidant at the tumor site. Active targeting of the delivery system can also be addressed by tuning the surface of the delivery system with targeting ligands (receptors that are overexpressed in cancer cell lines and not in normal tissue) so that chemotherapeutics can be delivered with high specificity, reducing the interaction with normal tissues (Figure 4).

Currently, most of the investigated antioxidants employed in anti-cancer treatments are at different stages of clinical trials (e.g., curcumin for the treatment of prostate cancer (NCT02064673) and resveratrol for the treatment of colon cancer (NCT00256334). Procyanidin, obtained from *Cinnamomi cortex* extract, can inhibit the regulated Nuclear factor erythoid 2-related factor 2 (NRF2) activity and NRF2 expression in human A549 NSCLC cells [35]. The flavonoid luteolin has also been reported to inhibit NRF2 in A549 NSCLC cells, decreasing cell proliferation and antioxidant gene transactivation [36]. Other phenolic compounds can promote antineoplastic activity despite exerting paradoxical activation on NRF2. Epigallocatechin gallate has been reported to promote chemosensitization to Cisplatin in TNBC MDA_MB231 cells and to inhibit tumor growth in xenografted mice by activating a NRF2-dependent antioxidant response with low side effects on normal tissues [37]. Other natural compounds, such as carnosol and resveratrol, were reported to act as chemopreventive agents by interfering with NRF2 activity by modifying intermolecular disulfide bonds between two KEAP1 molecules at Cys273 and Cys288, enhancing NRF2 nuclear accumulation [38].

Given the importance of the delivery systems in cancer (and other) treatments, we will review in the next sections the use of cyclodextrins, micro- and nanoemulsions, and liposomes as delivery systems of therapeutic antioxidants, highlighting different parameters that can affect their formation and stability in an attempt to provide the reader with a general understanding of their main characteristics and handling as versatile delivery systems. Some important considerations, including their commercial availability and their use to improve the beneficial properties of antioxidants (chemical stability, absorption rate, cellular absorption, and their bioactivity in cancer therapy), are also summarized.

### 3.1. Cyclodextrins as Delivery Systems of Antioxidants (AOs) with Anticancer Properties

**Figure 5:**
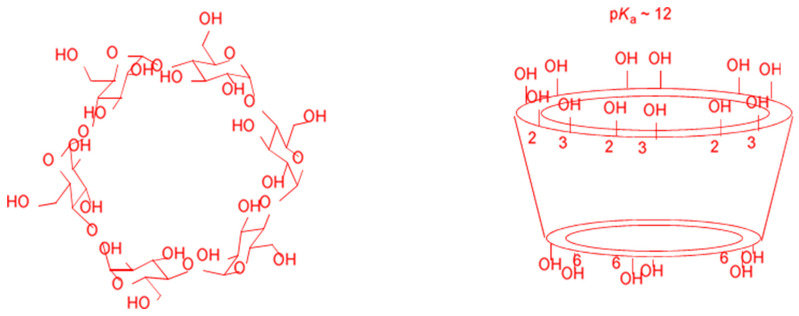
Chemical structure and conformation of natural cyclodextrins (CDs).

Cyclodextrins (CDs) are natural cyclic oligosaccharides composed of six, seven, or eight glucose units linked by α-1,4 glycosidic bonds (α-, β-, and δ-CD, respectively), Figure 5. They are formed from the enzymatic decomposition of starch from different sources, including potatoes and corn. Their spatial configuration resembles that of a hollow truncated cone, with the primary hydroxyl rim of the cavity opening having a smaller diameter than that of the secondary hydroxyl rim. The interior of the cavity has a polarity approximately half that of water, making CDs very attractive as host entities capable of hosting, in whole or in part, apolar molecules forming non-covalent host-guest inclusion complexes. The dimensions of their cavities are known, and as a rule of thumb, α-CD hosts substituted benzene molecules, β-CD, naphthalene molecules, γ-CD, and anthracene molecules. Some of the most important physicochemical properties of commercially available CDs are displayed in Table 2.

The formation of the CD-guest inclusion complex is usually described by means of the dynamic equilibrium indicated by Equations (1) and (2), highlighting that CDs can form complexes with different stoichiometry, most commonly 1:1, 1:2, and 2:1 (such as an antioxidant AO:CD), but also 1:3 and 1:4 complexes, although scarce, have been proposed. The inclusion constant *K*c is defined by Equation (2), and the higher the *K*c value is, the more stable the complex is and, thus, less susceptible to dissociation. Therefore, when this system is employed as a delivery system for antioxidants (AOs), the inclusion constants should be high enough to ensure their encapsulation but, at the same time, low enough to ensure their release.

(1) $$a\;AO+b\;CD\overset{K_{c}}{\;⇌}AO_{a}CD_{b}$$

(2) $$K_{c}=\frac{\left[{\mathrm{AO}}_{a}{\mathrm{CD}}_{b}\right]}{{\left[\mathrm{AO}\right]}^{a}{\left[\mathrm{CD}\right]}^{b}}$$

**Figure 6:**
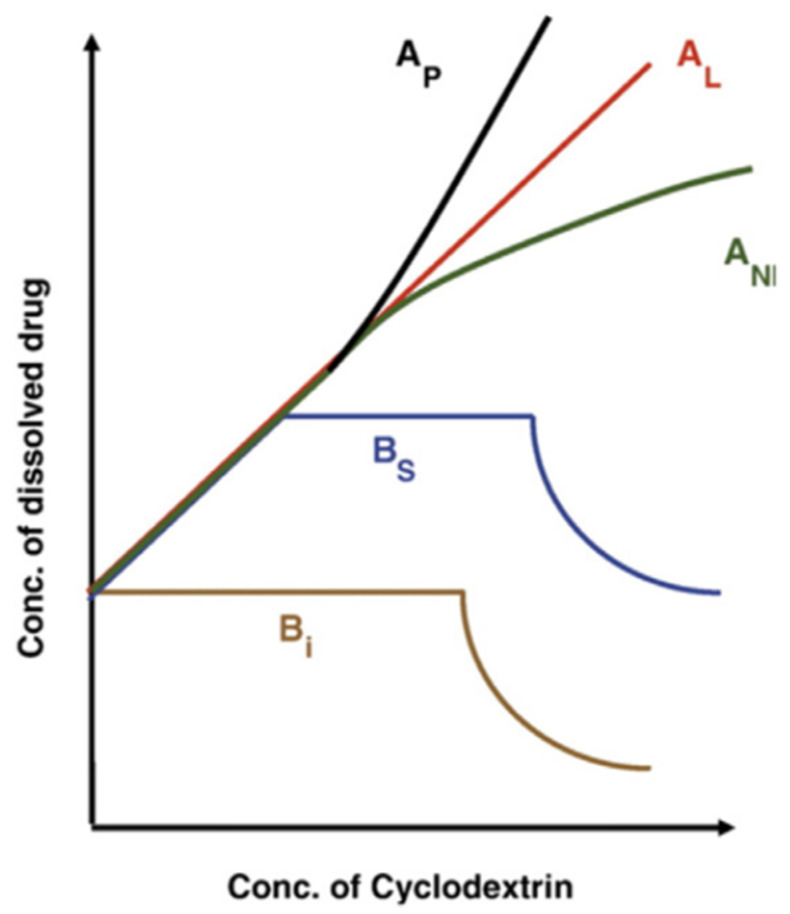
A typical representation of the A- and B-type phase solubility diagrams. Adapted from Martínez-Alonso et al. [41].

The values of the inclusion constants *K*_c_, Equation (2), can be easily determined from phase-solubility measurements. Experimentally, saturated solutions containing an excess of a poorly aqueous-soluble antioxidant (at least 3–4 times its solubility in water) are prepared in the presence of increasing concentrations of CDs. The flasks are shaken until equilibrium is reached at a constant temperature. The suspensions are then filtered, and the total concentration of the drug is calculated by employing an appropriate analytical technique (UV spectrophotometry, HPLC, etc.). According to Higuchi and Connors [39,40], the solubility profiles shown in Figure 6 are the most common. In the A-type profiles, the solubility of the guest molecule (i.e., antioxidant) increases upon increasing the concentration of CD, and it can be different: (1) in the A_L_-type, the solubility of the AO increases linearly with the concentration of CDs; (2) in the A_P_-type, a positive deviation from linearity is observed, probably due to the formation of higher-order complexes for CDs; and (3) in the A_N_-type, which is more difficult to interpret, a negative deviation from linearity is observed, suggesting a potential self-association or aggregation of the CDs or their complexes and then decreasing the antioxidant solubility. In the case of B-type phase solubility profiles, the formation of AO-CD complexes with limited solubility is observed in the aqueous complexation environment. The Bs-type profile shows the formation of poorly aqueous-soluble complexes that obtain the highest solubility at the plateau. For Bi-type profiles, the obtained complexes have low aqueous solubility in the studied solvent.

For the formation of a 1:1 inclusion complex, a representation of the antioxidant concentration in solution against the concentration of CD should be an A_L_-type phase solubility diagram where the intercept defines the intrinsic drug solubility (S_0_). It is possible to determine the inclusion constant Kc from the slope of the straight line according to the equation described by Higuchi and Connors [40], Equation (3), where S_0_ is the concentration of the drug without CD.

(3) $$K_{1:1}=\frac{\mathrm{slope}}{S_{0}\left(1-\mathrm{slope}\right)}$$

From the discussion above, it follows that CD-guest complexes employed to deliver antioxidants must fulfill two main conditions: (1) they must show an adequate complexation of the guest molecule to be transported efficiently to the specific disease site, and (2) an optimal conjugation of targeting moieties on the CD scaffold. It is valuable, therefore, to get some auxiliary knowledge on the physicochemical properties of antioxidants, on their pharmacokinetic profile, on their biochemical characteristics at target sites, and on relevant clinical requirements.

The selection of a particular CD for a specific drug delivery application depends on different characteristics, such as its solubility, biocompatibility, and administration route. β-CD is the most common CD employed in pharmaceutical formulations and is not toxic after oral administration. This is the case for most of their derivatives. On the other hand, while the derivatives HP-β-CD and SBE-β-CD have been approved for parenteral use, β-CD, α-CD, and methylated β-CDs are not appropriate for parenteral administration because of their reported renal toxicity. δ-CD is not employed for intravenous administration due to the fact that it could form aggregates in aqueous solutions [42]. Once the host molecule has been chosen, the formation of AO-CD complexes may result in a valuable multifunctional technology with various benefits, including: (1) improvement of the solubility of poorly soluble antioxidants, their dissolution, and bioavailability; (2) minimization of unpleasant tastes and smells; (3) preventing interactions of the antioxidant with other solution components; (4) prolongation of the shelf life of antioxidants; their physical, chemical, and thermal stability; (5) modification of the antioxidant release sites; and (6) easy functionalization of the surfaces of CDs, i.e., tailored ligands can be linked to CDs to facilitate the CD-guest complex to reach the surface of target cell [43]. Such multi-functionality makes CDs attractive excipients for their use in the preparation of new anti-cancer formulations or in the reformulation of existing ones. Table 3 shows some of the investigated CD-based delivery systems that are still in the early testing phases before clinical trials. The introduction of a third component in the solution (e.g., polymers, organic acids, metal ions, or lipids) can boost the formation of AO-CD complexes. For example, Mazyed et al. [44] developed a noisomely functionalized HP-β-CD/6-gingerol complex, increasing its permeability and stability while at the same time providing a potential use of secondary metabolites for treating cancer diseases.

As discussed above, the main drawbacks to the use of natural antioxidants with potential anticancer properties lie in their low solubility and bioavailability. This limitation can be eventually overcome through the formation of inclusion complexes. A literature survey indicates that CD-encapsulated therapeutic substances are capable of reducing tumor growth with minimal doses of bioactives, improving antioxidant solubility, and increasing bioavailability. It has also been reported that such CD-guest complexes may also modify the pharmacokinetic parameters of the active substances in diverse animal cancer models compared to free drugs alone.

**Figure 7:**
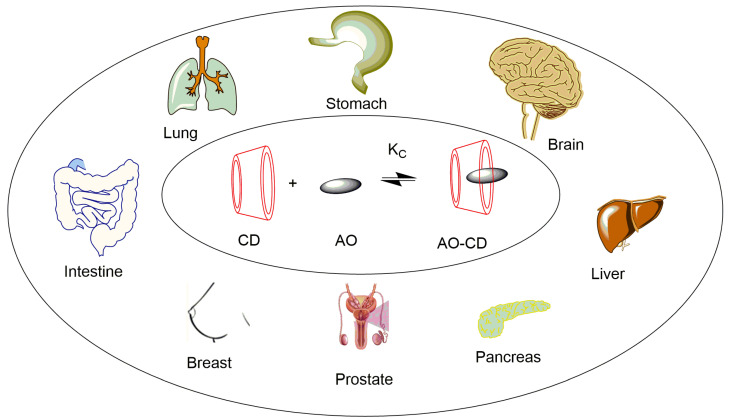
Some applications of cyclodextrin complexes in cancer treatment (AO: antioxidant or another anticancer drug).

To sum up, the complexation of anticancer agents with CDs may produce a new generation of active therapeutics with improved potential for cancer treatments. Figure 7 displays some of the cancer diseases where host-guest technology is being explored.

### Antioxidants Are Commonly Employed for Effective Cancer Prevention and Therapy

**Figure 8:**
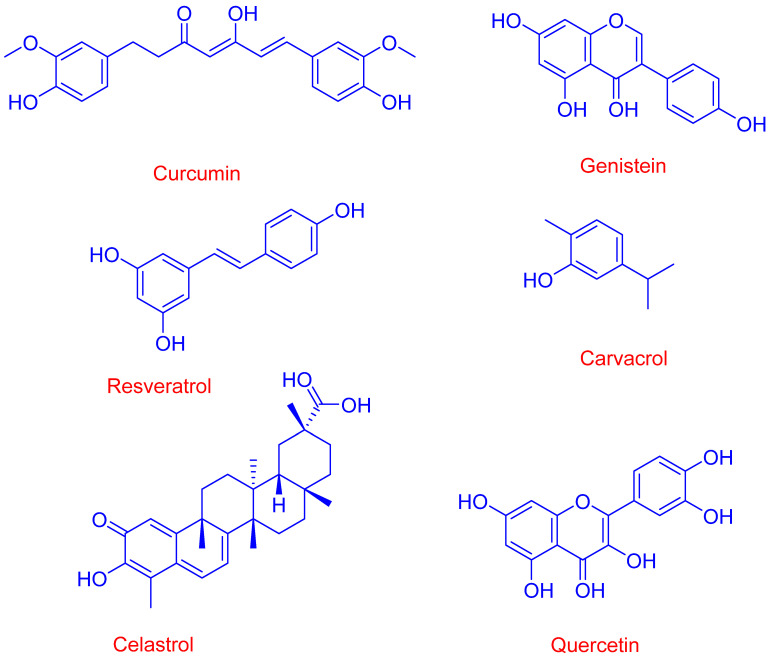
Chemical structure of representative natural antioxidants (AOs) with anticancer properties that can be enhanced when complexed with cyclodextrins.

Various antioxidants have been involved in the fight against cancer, with different degrees of efficacy. Figure 8 shows the chemical structures of some of them.

Curcumin (1,7-bis(4-hydroxy-3-methoxyphenyl)-1,6-heptadiene-3,5-dione) is a yellow-colored polyphenol derived from *Curcuma longa Linn* that is employed in the treatment of different diseases because of its antimicrobial, anti-inflammatory, antioxidant, anticancer and neuroprotective effects [50]. Curcumin is poorly soluble in aqueous solutions because it is very hydrophobic; however, different studies have evaluated the solubility and bioavailability of curcumin-CD complexes [50], reporting a significant improvement in the curcumin solubility (up to 10,000-fold) compared to that for the uncomplexed (free) compound [51]. Patro et al. reported improved bioavailability for different curcumin-CD complexes, which was higher for curcumin-α-CD complex than that observed for curcumin-γ-CD complex [52]. In a different study, it was reported that curcumin-HP-β-CD complexes could improve cellular uptake and increase the half-life of curcumin in different cancer cell lines when compared with those of the uncomplexed bioactive [53]. The developed curcumin-cyclodextrin complexes seem a viable solution for enhancing their solubility and bioavailability by crossing biological barriers (e.g., the tumor microenvironment), as shown in Figure 8. Cavacurmin^®^, an approved γ-cyclodextrin-based formulation of curcumin, has shown enhanced bioavailability (85-fold compared to that observed for the free antioxidant) in a crossover study [54].

The anticancer activity of curcumin is attributed to its ability to promote apoptosis and reduce tumor proliferation via regulation of different biochemical and signaling pathways [50]. Yallapu et al. [55] found that the cellular uptake of curcumin-CD complexes improved in DU145 prostate cancer cell lines when compared to that of the free bioactive. Curcumin complexes decrease the size of tumors in a xenograft mouse model of lung cancer [56] and Kazemi-Lomedasht [57] have reported that the curcumin-β-inclusion complex shows improved curcumin uptake in T47D breast cancer cells and an important decrease in the level of telomerase gene expression in comparison to cells treated with the free compound. It was also reported that curcumin-CD complexes minimize the rates of cancer cell migration by 75.5% (a two-fold increase when compared with that of free curcumin) and enhance apoptosis rates by 26.3% (again, a two-fold increase compared to that of free curcumin) [58].

Curcumin-CD complexes have also been developed for theragnostic purposes (treatment and diagnosis). Yallapu et al. [55] developed a platform (an iron oxide core coated with β-CD and Pluronic F68) for encapsulated curcumin, reporting significant anticancer properties compared to the free compound. Curcumin nanoparticles enhanced the production of reactive oxygen species, promoting apoptosis in MDA-MB 231 cancer cells.

**Figure 9:**
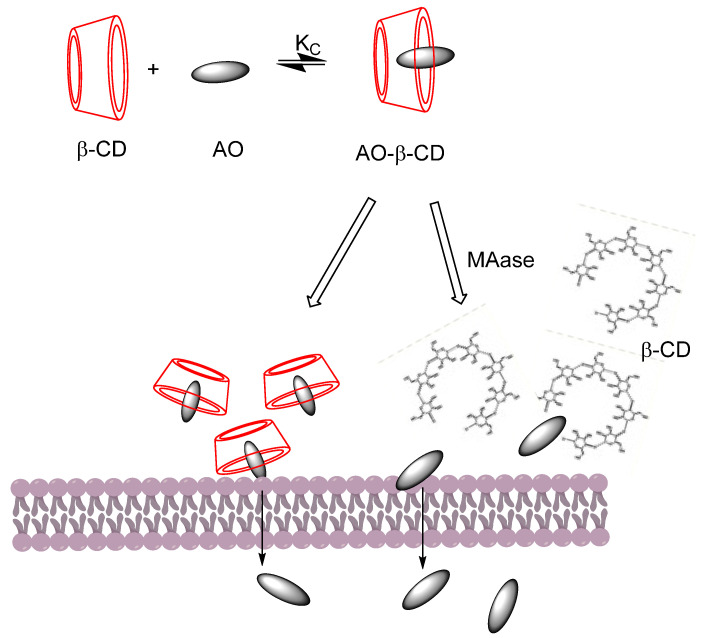
Release of the AO-CD complex in a controlled manner in the absence and presence of maltogenic amylase (MAse). A faster release of curcumin was reported by Sahar Roozbehi et al. [60] in the presence of MAse. Adapted from reference [60].

Combinations of curcumin-CD complexes with other agents for treating cancer diseases have been explored by Rocks et al. [56], who found that curcumin-CD complexes enhanced the therapeutic activity of gemcitabine in an experimentally induced model of lung cancer. Synergistic effects on different cancer models of the ovarian, prostate, lung, and breast were reported when curcumin and paclitaxel (a chemotherapy taxane drug) were encapsulated with poly(β-cyclodextrin triazine) [59]. Another study proved that curcumin can be released in a controlled way in the presence of maltogenic amylase by preparing an enzyme-triggered drug delivery system employing β-CD (Figure 9). Results revealed an enhanced uptake of curcumin and an apoptotic influence in different cancer cell lines [60].

Resveratrol (3,5,4′-trihydroxystilbene) is a polyphenol whose anti-inflammatory, anti-tumor, anti-aging, antioxidant, and anticancer properties have been described in the literature [61,62]. Resveratrol was reported to participate in several molecular pathways, including tyrosine kinase, phosphoinositide-3-kinase (PI3K)/AKT.STAT3/5, and nuclear factor kappa N (NF-kB) pathways. Resveratrol directly interferes with different states of carcinogenesis (initiation, promotion, and progression). In vitro experiments, the anti-proliferative and pro-apoptotic activities of resveratrol were suggested to be due to its ability to modulate the expression of pro-oncogenic and tumor suppressor microRNAs, the peroxisome proliferator-activated receptor, nuclear respiratory factors (NRF-) 1 and 2, nuclear factor-kappa B (NF-kB), and gamma activator 1 alpha (PCG-1α) among others.

Detrimentally, resveratrol is a poorly water-soluble compound (aqueous solubility ~0.03 mg/mL [60]), limiting its direct use as a therapeutic agent and its benefit to health. Its aqueous solubility can, however, be significantly improved (up to 1.1 mg/mL at T = 25 °C) by formulating resveratrol in the form of inclusion complexes with CDs. The formation of an inclusion complex can also increase its chemical stability and its permeation ability across biological membranes, enhancing its retention effect (EPR) in tumor sites [63]. Positive effects were also reported when analyzing its anticancer activity in vitro on human breast cancer cell lines (MCF-7) [63]. Berta et al. [64] studied the influence of the resveratrol complexed form with 2-hydroxypropyl-β-cyclodextrin in induced hamster oral squamous cell carcinoma cell lines (HCPC I) and in an animal model. The results showed a mitigation in oral lesions (dysplasia, hyperplasia, and carcinoma) and a reduction in the incidence of squamous cell carcinoma. Lu et al. [65] investigated the cytotoxicity of resveratrol/β-cyclodextrin and resveratrol/2-hydroxypropyl-β-cyclodextrin complexes in cancer (He1.a and Hep3B) in healthy human umbilical vein endothelial cell [HUVEC] lines. The authors concluded that the CD-complexes showed a significant level of cytotoxicity in both cancer cell lines and had a preventive effect on healthy cells.

Other natural antioxidants have also gained wide interest due to their variety of therapeutic activities (antimicrobial, antioxidant, anti-inflammatory, and anticancer) when complexed with cyclodextrins. Ameeduzzafar Zafar et al. [66] prepared a genistein inclusion complex by employing 2-hydroxypropyl-β-cyclodextrin and poloxamer 188 to improve its aqueous solubility and in vitro activity. Their results demonstrated higher in vitro antioxidant and cell viability activity in the MCF-7 breast cancer cell line than that observed for free genistein.

Quercetin and rutin inclusion complexes were prepared with HP-β-CD, and the results showed dose-dependent cytotoxicity even on Human Breast Adenocarcinoma (MDA-MB-231) and Human Lung Carcinoma cell lines (A549 cells) [67]. Celastrol, an active phytochemical constituent, is extracted from *Tripterygium wilfordii* and has also shown promise in in vitro studies for treating cancer diseases. In a study reported by Shukla et al. [46], a sulfobutyl-β-CD complex of celastrol was developed and evaluated for its in vitro anticancer activity on human lung cancer cells. The results revealed improved cytotoxic activity compared to the free compound. β-cyclodextrin complexes with carvacrol (a monoterpene derived from thyme oil) exerted a dose-dependent reduction of tumor cell growth in the prostate cancer cell line (PC3) in vitro [49]. Ke et al. [68] prepared a γ-cyclodextrin-based metal–organic framework (CDMOF) loaded with epigallocatechin-3-gallate, showing strong cancer cell growth inhibitory effects on C6 cells.

Despite cyclodextrins (CDs) attracting increasing interest over the past few years, leading to promising antitumor nanomedicines, further research on CD-based therapeutic formulations against many types of cancer is needed to take advantage of their chemical structures, ease of chemical modifications, natural origin, biocompatibility, low immunogenicity, and commercial availability.

### 3.2. Microemulsion/Nanoemulsion as Delivery Systems of Antioxidants with Anticancer Properties

During the last few decades, emulsified-based delivery systems, including microemulsions, emulsions, and double emulsions, have emerged as prospective systems for drug delivery. The reasons for this are various. Emulsions, nanoemulsions, and microemulsions are considered excellent delivery systems to transfer antioxidants (and other drugs) to target sites, improving their bioavailability and minimizing their undesired action on other cells or tissues. These systems have been successfully employed in cancer therapy, overcoming the low aqueous solubility of bioactives while also enhancing their bioavailability by improving their bioactive transport to cancer cells. Some of the reported advantages of emulsions as carrier systems include:

- Improvement of the thermodynamic stability of antioxidants;
- Better solubility of lipophilic antioxidants;
- Masking of undesirable tastes;
- Improvement of the bioavailability and biodegradability of antioxidants;
- Reduction in the toxicity of antioxidants;
- Enhancement of the drug release profile.

**Figure 10:**
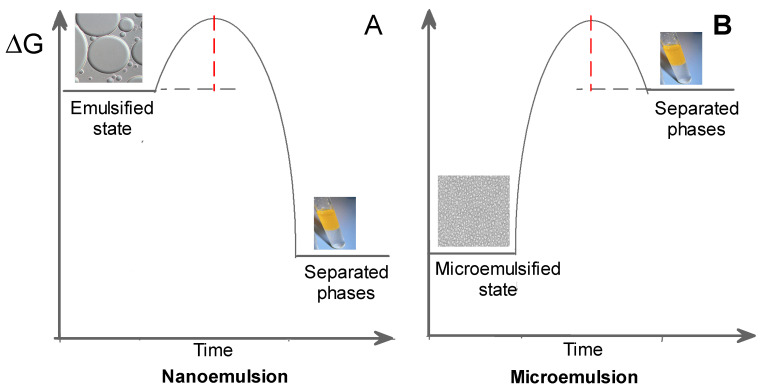
Energy difference between nanoemulsion (**A**), microemulsion (**B**), and separated phases. Microemulsions are thermodynamically stable systems, whereas emulsions or nanoemulsions are unstable thermodynamically because the Gibbs free energy of the emulsified system is much higher than the sum of the Gibbs free energies of the individual components.

Microemulsions and nanoemulsions are composed of two immiscible liquids, one dispersed in the other in the form of small droplets (Figure 10), and can be classified, depending on the nature of the continuous phase, as water-in-oil (*W*/*O*, water dispersed in oil) or oil-in-water (*O*/*W*, oil dispersed in water) systems. The most important difference between microemulsions and nanoemulsions is their thermodynamic stability. Microemulsions are thermodynamically stable, transparent, dispersions of the two immiscible liquids, a surfactant (and sometomes a cosurfactant), is needed to stabilize microdomains, reducing the interfacial tension between the two immiscible liquids. On the contrary, emulsions are thermodynamically unstable and tend to separate spontaneously (Figure 10).

The preparation of microemulsions and nanoemulsions needs similar ingredients: an oil phase, an aqueous phase, and a surfactant (and, if necessary, a co-surfactant too). Both emulsified systems can be easily prepared by employing non-toxic, biocompatible, and biodegradable ingredients and should meet the FDA guidelines.

The oil phase commonly contains a combination of long-, medium-, and short-chain triglycerides (LCT, MCT, and SCT, respectively) from natural vegetable sources: olive, soybean, cottonseed, sesame, coconut, and rice bran oils, among others. Oleic acid and ethyl oleate have frequently been employed in the preparation of oral, parenteral, and topical emulsions. Efforts are being undertaken to incorporate omega-3-rich marine oils, which are rich in polyunsaturated fatty acids and provide therapeutic benefits, because they are convenient and safe alternatives for atherosclerotic patients who cannot tolerate regular LCTs and MCTs. Unsaturated oils are, however, prone to oxidation and the purity of the oil phase and the absence of dangerous components such as lipid peroxides need to be controlled.

The aqueous phase can contain ionic or osmotic agents, hydrophilic antioxidants (e.g., ascorbic acid), buffers, and preservatives (ethylenediaminetetraacetic acid (EDTA), sodium benzoate, benzyl alcohol, etc.). Other components that may be included are antimicrobial agents such as the methyl and butyl derivatives of p-hydroxybenzoic benzalkonium chloride and parabens [69].

**Figure 11:**
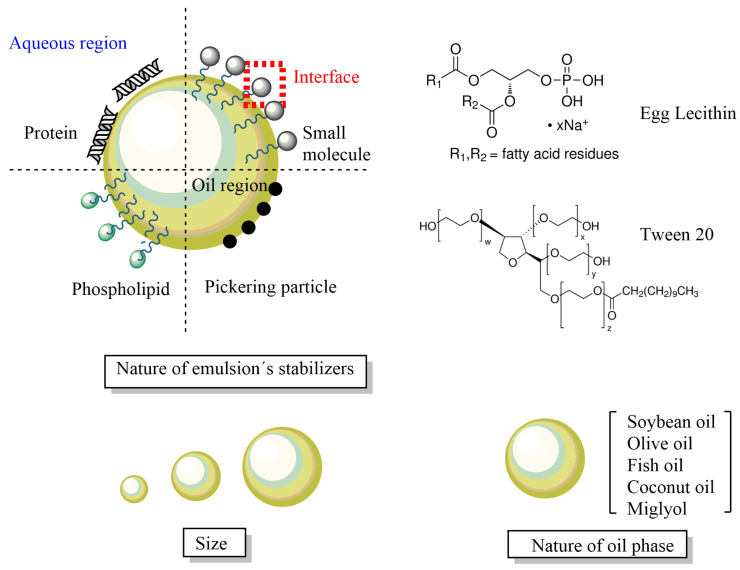
Schematic representation of different types of emulsified delivery systems. The applications and properties depend on the nature of the surfactants employed to stabilize them kinetically, the nature of the oil, and the molecules and ions dissolved in the aqueous phase.

To stabilize the emulsions kinetically, amphiphilic molecules (surfactants) that adsorb at the oil-water interfacial region provide steric and/or electrostatic stabilization. Surfactants may be non-ionic, cationic, anionic, or zwitterionic, as shown in Figure 11. Food-approved surfactants from natural sources include lecithin (phosphatidylcholine), derived from egg yolk or soybean, and amphiphilic proteins such as casein, β-lactoglobulin, and polysaccharides (e.g., gums and starch derivatives). Synthetic surfactants such as Tweens (sorbitan monolaurate) and Spans are also frequently employed (Figure 11).

The most common emulsified system used in pharmaceutics is of the type *O*/*W*, so that the oil is present in the form of small droplets surrounded by the continuous aqueous phase. The diameters of the droplets range from 10 (nanoemulsions) up to several hundred (macroemulsions). The size of the droplet is a critical parameter that affects not only the physical stability of the nanoemulsion (the smaller the diameter, the higher the physical stability) but also the application of the delivery system. For example, emulsified delivery systems with droplet diameters greater than 5 µm can be confined in the lungs, causing a pulmonary embolism [70]. When employed to deliver drugs in the form of injectable emulsions, the diameters of the droplets should be lower than 500 nm; otherwise, they do not work and may even be dangerous [71].

**Figure 12:**
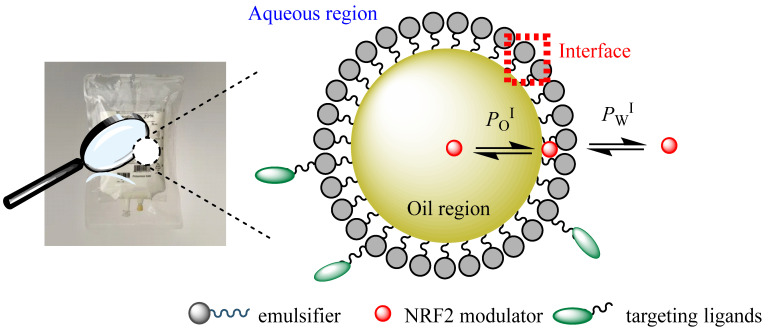
In the absence of physical barriers, drugs distribute thermodynamically between the different regions of a micro-/nanoemulsion according to their solubility in each region [13,73].

Both nanoemulsions and microemulsions can be conceptually divided into three regions with different solvent properties: the aqueous, interfacial, and oil regions, as shown in Figure 12. When loaded with, for instance, antioxidants (or another drug) for delivery, the antioxidant distributes thermodynamically between the three regions according to its polarity. Hydrophilic antioxidants will be mostly located in the aqueous and interfacial regions, while relatively apolar antioxidants will distribute preferentially between the oil and interfacial regions. This generates emulsified systems to enhance the solubility of hydrophobic drugs and their bioavailability [72]. Figure 12 illustrates the distribution of the NRF2 modulator between the different regions of an emulsified system.

The distribution of the antioxidant between the different regions of the emulsified system is crucial to carrying out a therapeutic action. For example, Takino et al. [74] reported that the in vivo availability of drugs with log *p* > 9 (*p* is the octanol-water partition constant *P*_W_^OCT^, defined as the ratio of the effective concentrations of the drug in the octanol and water phases (moles of antioxidant per unit volume of each phase)) can be modulated by modifying emulsion properties. Nevertheless, drugs with lower log*P* values (e.g., cinnarizine (log *P* = 5.8), chlorambucil (log *P* = 1.7), and docetaxel (log *P* = 5.8)) have also been successfully employed in emulsified systems to enhance their pharmacokinetic properties [75].

#### Some Antioxidant-Loaded Nanoemulsions Employed in Cancer Diseases

A growing interest has been focused on the nanoformulation of delivery systems loaded with naturally occurring bioactive compounds for chemotherapy and chemoprevention in different types of cancer, leading to improved permeability and retention (EPR) effects, enabling several payloads, and minimizing side effects.

The solubility, bioavailability, and efficiency of fisetin, a flavonoid, can be improved when it is administered as a nanoemulsion formulation. According to the results obtained by Héloïse Ragelle et al., the bioavailability of fisetin was increased by 24-fold when it was administered intraperitoneally in mice. In addition, fisetin exerts anticancer activity on lung carcinoma-bearing mice at ~5–6 smaller doses in comparison to the free compound [76]. A low dose, such as 36.6 mg/Kg of fisetin loading in a nanoemulsion formulation, decreased the tumor volume by 53%, while a dose of 223 mg/kg is required for free fisetin, as reported in another study [77].

Lycopene has been attracting a lot of interest in recent years due to its anticancer activity and the fact that it can be used to treat different cancer diseases. Its anticancer activity is suggested to be due to its efficacy in triggering the cell cycle block of hepatoma cells, lung cancer cells, and breast cells, the reduction of the propagation of colon cancer cells and leukemia cells, and the antimetastasis of hepatoma cells. However, its low bioavailability minimizes its application for treating a variety of cancer diseases. Huang et al. [78] prepared a nanoemulsion carrier system loaded with lycopene and gold nanoparticles and evaluated their anticancer activity in a HT-29 colon cancer cell line. The inclusion of gold nanoparticles with selective cancer cell receptor ligands can improve the specificity and efficacy of chemotherapeutic antioxidants. The results showed synergistic effects for the combined treatment with a lycopene-nano-gold nanoemulsion against colon cancer [78]. The major diffusion of the nanoemulsion formulation into the cytoplasm and nucleus suggests that it can be a potential carrier system for passive targeting through the EPR effect. Modulating the processes controlled by the nucleus is an attractive strategy in nuclear-targeted cancer therapy. However, the authors suggested that more in vitro and in vivo experiments are needed to understand the underlying mechanism.

**Figure 13:**
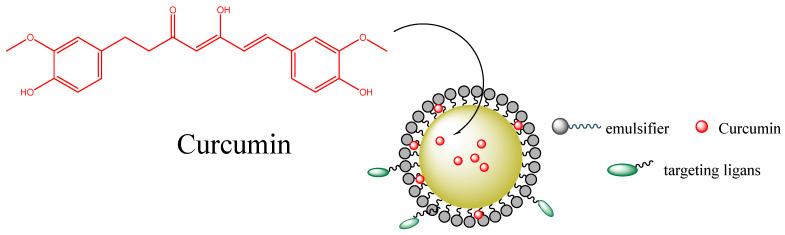
Curcumin-loaded nanoemulsions and their potential distribution between the different regions of the emulsified system [84].

*In vitro* studies have also revealed the anticancer activity of tocotrienol nanoemulsion-based formulations containing anticancer therapeutics [79]. Likewise, tocotrienols, found in palm oil and rice bran oil, play an important role in cancer therapy by modulating growth factors (EGF-dependent PI·K pathway and TGF-β) or through pro-apoptotic, anti-metastatic, and anti-angiogenesis properties. A synergistic effect was observed for the combination of a curcumin-loaded δ-tocotrienol nanoemulsion evaluated in human cancer cells such as MCF-7 and OVCAR-8 [80]. Several in vitro and in vivo studies suggested that a nanoemulsion could improve the anticancer activity of curcumin, as shown in Figure 13. For example, in another study, a gelatin-encapsulated nanoemulsion containing curcumin was evaluated for its anticancer activity against MDA-MB-231 breast cancer cells and showed a significant ability to stabilize curcumin and increase its bioavailability in comparison to a free compound [81]. Machado et al. [82] reported that the employment of curcumin loaded nanoemulsion formulations showed a high phototoxic effect, minimizing the proliferation of cells and improving the generation of reactive oxygen species in a human breast adenocarcinoma cell line and a human foreskin fibroblast cell line. Guant et al. [83] also evaluated the anticancer activity of curcumin-loaded nanoemulsions, which exhibited a higher cytotoxicity than that observed for free curcumin for prostate cancer. Other curcuminoids (desmethoxy curcumin and bisdesmethoxy curcumin) loaded in nanoemulsions also showed good cytotoxic activity, supporting their potential application in prostate, colorectal, leukemia, and glioblastoma cancers.

Piplartine nanoemulsion formulations provide the ability to improve oral bioavailability by increasing it 1.5 times in comparison to the free compound. Nanoemulsions loaded with Piplartine showed important antitumor activity against melanoma in mice [85]. The prepared nanoemulsion significantly improved the bioavailability of Piplartine and reduced the growth of melanoma tumors in vivo in comparison to free Piplartine. In this study, it was observed that there was an important increase in the concentration of Piplartine in plasma when it was administered in a nanoemulsion form as compared to a free compound.

Nanoemulsions containing Epigallocatechin-3-gallate have also shown a significant effect on the inhibition of a H1299 lung cancer cell line and differ from the activity of a free compound on adenocarcinoma cells. By employing a nanoemulsion carrier, lung cancer cell colony formation, migration, and invasion were minimized in a dose-dependent manner by activating the AMPK signaling pathway. It can also reduce lung cancer cell invasion following metalloproteinase (MMP)-2 and MMP-9 independent mechanisms [86].

Nanoemulsion formulations have also improved the anticancer properties of both Cisplatin and Quercetin tested against breast cancer MDA-MB-231 and renal HEK-293 cells. Results also showed the synergistic effect of Quercetin/Cisplatin against MDA-MB-231 [87].

Resveratrol has excellent chemotherapeutic and chemopreventive properties against different cancers. It has been reported that resveratrol promotes the apoptosis of tumor cells, inhibits tumor-derived nitric oxide synthase expression and cyclooxygenase (COX) activity, and prevents tumor growth and migration [88]. Resveratrol-loaded nanoemulsions have been shown to exert a significant increase in in vitro cytotoxicity in MCF-7 breast cancer cells. Likewise, this study revealed improved antiangiogenic activity in an in vivo chick chorioal-lantoic membrane assay [89].

## 4. Controlled Drug Delivery Systems

**Figure 14:**
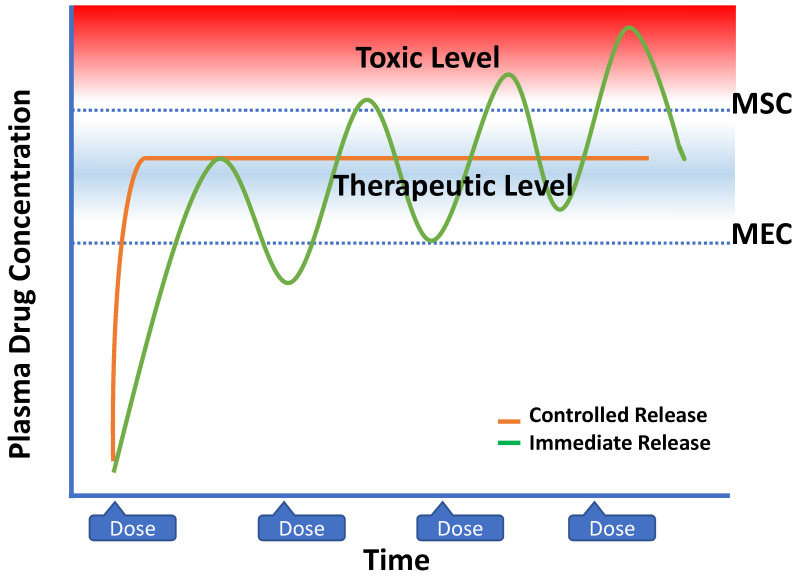
Plasma drug concentration profiles from zero-order controlled-release dosage forms and multiple dosing of conventional dosage forms. (MSC: maximum safe concentration, and MEC: minimum effective concentration).

Typically, the conventional or immediate-release dosage forms are administered at a high dose at a given time, and repeated drug administration is required for effective therapy. This form of treatment reduces the controllability of therapy due to reasons such as missing or repeated medicine intake, inadequate blood drug levels, and/or increased side effects. The main problem that arises with the frequent and repeated administration of conventional dosage forms can be related to the drug concentration rising above the toxic level or falling below the effective level (Figure 14). Thus, the challenges mentioned have led researchers to the discovery of modified release dosage forms. According to the European Medicines Agency (EMA) Guidelines on the Quality of Oral-Modified Release Products, modified-release dosage forms are defined as preparations that release their content at a different rate and/or region than conventional dosage forms administered via the same route [90]. Drugs that provide prolonged release, delayed release, pulsatile release, and accelerated release are included in this category. Due to the different release characteristics, several different expressions are used to describe controlled release systems, and different types of classifications are found in references.

Controlled drug delivery systems (CDDS), which typically provide continuous drug release for a prolonged period of time in the desired zone and extend the systemic absorption and biological activity of the active agents, have numerous advantages regarding therapeutic effects and patient compliance [91]. However, there are several formulation and cost-associated limitations that should be considered while designing controlled drug delivery systems (Table 4) [92,93].

### 4.1. Development of Controlled Drug Delivery Systems

Controlled drug delivery systems (CDDS) are used especially in the treatment of chronic diseases (cancer, hypertension, etc.) and other conditions (contraception, etc.) that require repeated drug administration for a long period of time. The introduction of CDDS to the drug market was in 1952 with SmithKline and the French product Dexedrine, which provided a 12 h dextroamphetamine release profile. Subsequently, the first generation of CDDS has been developed by using different mechanisms, including dissolution, diffusion, osmosis, and ion exchange. These systems have generally been developed to overcome the physicochemical problems of Active Pharmaceutical Ingredients (API) and are administered as oral and transdermal formulations. Since the 1980s, second-generation controlled drug delivery technologies have been introduced, focusing on the development of zero-order drug release and overcoming biological barriers [94]. Nanoparticle-based drug carriers, peptide-protein delivery, and the usage of smart polymers for CDDS have been major interests of this second-generation period, which lasted between 1980 and 2010. The third-generation drug delivery systems remarkably focus on overcoming the formulation (poor aqueous solubility, burst drug release of depot systems, etc.) and biological (gastric retention time, site-specific absorption, targeting of specific tissues, etc.) barriers that prevent efficient drug therapy. Modulated delivery systems such as glucose-sensitive insulin-releasing systems, surface-decorated targetable nanodrug carriers, and many others have shown promising results [94,95]. Controlled drug delivery systems differ from sustained-release drug delivery systems in that they necessarily provide zero-order drug release.

**Figure 15:**
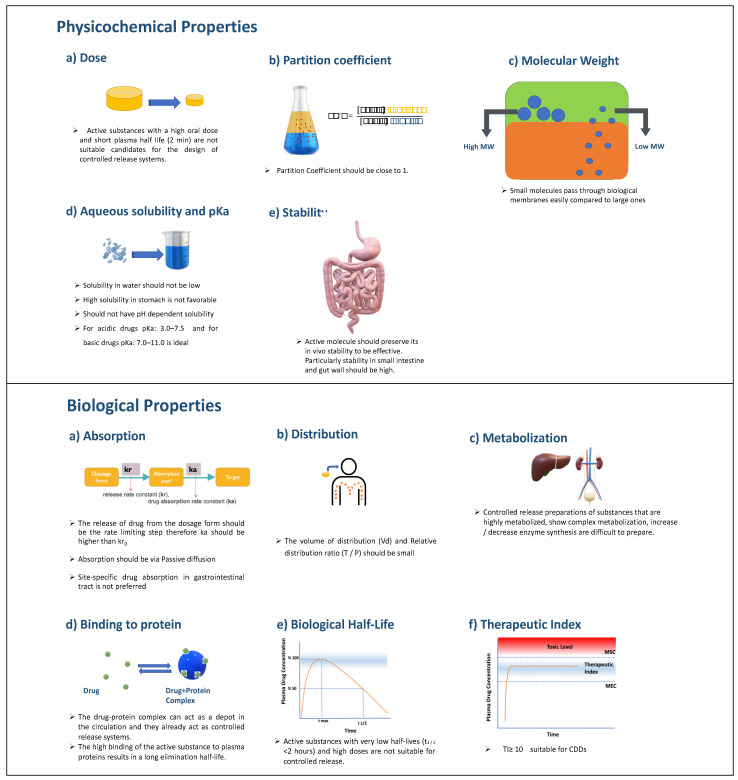
Critical physicochemical and biological properties that should be evaluated while designing a CDDS.

A constant drug release rate from the dosage form plays a major role in achieving adequate drug concentration in the blood or specific tissues. The API to be absorbed must first dissolve in the aqueous phase in the region of application and then pass through the biological membranes. For CDDS, the blood drug level must be determined by the dissolution rate of the drug, not physiological factors. Therefore, the release rate constant (*k*r) should be less than the drug absorption rate constant (*k*a). While designing CDDS, one should consider both the biological and physicochemical properties of active agents. After examining these parameters, which are presented in Figure 15, the target drug release profile for the drug should be determined, and R&D studies should be carried out in this direction [96].

### 4.2. Polymers for Controlled Drug Delivery

Different polymers are used to enable controlled drug release in CDDS. Recent advances in polymer technology have led to the emergence of a wide variety of polymeric materials that can be used in CDDS. Polymers can be classified based on origin source (natural, synthetic, and semi-synthetic); structure (linear, branched, and cross-linked network); polymerization technique (addition polymers and condensation polymers); and biodegradability (biodegradable and non-biodegradable) [97,98]. Under in vivo conditions, chains of biodegradable polymers are broken down by hydrolytic or enzymatic means, resulting in natural byproducts such as oxygen, nitrogen, carbon dioxide, and water. This is particularly useful for implants, as surgical removal of the implant is not required after drug release is complete. Therefore, water-soluble and water-insoluble biodegradable polymers are often used in CDDS. Examples of the polymers used in CDDS are listed in Table 5 [31,99,100,101,102].

### 4.3. Mechanisms of Drug Release from CDDS

**Figure 16:**
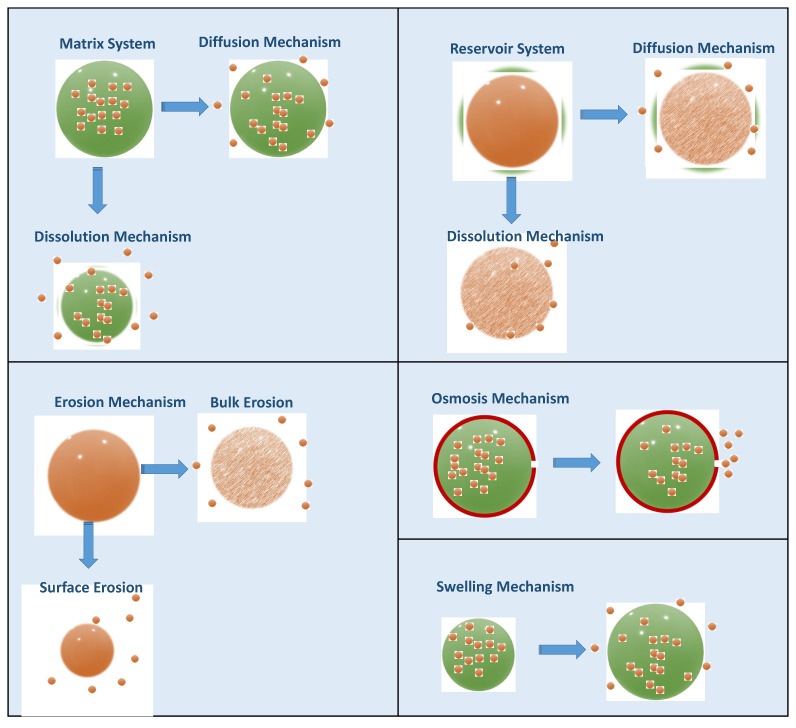
Different mechanisms of drug release from CDDS.

Depending on the design of the CDDS, the API can be released by different mechanisms, including dissolution, diffusion, osmosis, chemical degradation, and swelling (Figure 16). Dissolution-controlled drug release can be observed in both matrix and reservoir systems. Matrix systems are formed by homogenously dispersing the API in a polymeric material, and in reservoir systems, the drug core is surrounded by a polymeric membrane. In these systems, the solubility of the polymer is critical for controlling the drug release rate. In order to control the dissolution rate of highly water-soluble compounds, a slowly soluble polymeric matrix material (e.g., waxes) can be used. In reservoir systems, after the dissolution of the polymeric membrane, the drug is released. Therefore, solubility and thickness of the membrane are critical release rate-control parameters [96]. In diffusion-controlled systems, the efflux of drug molecules from CDDS to the dissolution medium is directed by the concentration gradient of the API [103]. Diffusion can occur through a polymeric membrane or polymeric matrix. This drug release profile is explained by Fick’s first law, and parameters such as temperature, pH, polymer type, and molecular weight of the API may alter the diffusion rate [104]. In the CDDS that work with the principle of osmosis, water enters the system through a semi-permeable membrane, dissolves the API, and releases through the orifice on the dosage form until the osmotic pressures on both sides are equal. In biological media, CDDS containing biodegradable polymeric materials release their contents via chemical degradation of the polymer. This mechanism is also called erosion, and there are two different erosion mechanisms in CDDS: bulk erosion and surface erosion. The chemical structure of the polymers, including molecular weight configurations, may alter the erosion mode and drug release rate [21]. Swellable polymers, which have a rigid structure in dried form, can swell as they come into contact with the aqueous biological medium. During this swelling process, API is released through the swelled matrix of the CDDS. Other than these typical drug release mechanisms, different approaches, such as ion exchange resins, stimuli-sensitive systems, and gastroretentive systems, are being developed for controlled drug release.

Controlled release technology is used in many different dosage forms, including tablets, capsules, implants, transdermal therapeutic systems, stents, etc. CDDS can be administered via oral, parenteral, transdermal, respiratory, intravaginal, intrauterine, and nasal routes. As mentioned in this section, APIs that are rapidly eliminated from the body, undergo physiological degradation before absorption, and have a narrow therapeutic index are not qualified as suitable CDDS candidates. Unfortunately, APIs with these features are often eliminated early in the discovery and development phases. However, the efficiency of these molecules can be increased by drug targeting. This approach has led to breakthrough nanotechnology-based drug carriers (nanoparticles, micelles, liposomes, etc.) in the last three decades. With a better understanding of the mechanisms and technologies of controlled release, it will be possible to convert such APIs into final products [105].

The recent progress of CDDS has significantly increased the number of marketed controlled-release drug products and their popularity (Table 6).

### 4.4. Thermosensitive Smart Liposomes for Cancer Chemotherapy

Most anticancer agents have poor aqueous solubility and low in vitro and in vivo stability, which shorten the plasma residence time [106]. Poor tumor specificity, high toxicity in healthy tissues, low bioavailability, and drug resistance are several other issues that must be considered in order to improve cancer therapy. The need for new treatment paradigms in cancer therapy led researchers to concentrate on the development of potential drug delivery systems. Particularly, liposome-based drug delivery systems have opened new horizons in this field and have been approved for clinical use in oncology. Liposomes are vesicular nanocarriers composed of biodegradable and biocompatible lipids with the capability of encapsulating various types of drugs. Liposomes offer the advantages of good biocompatibility, high drug encapsulation efficiency, and improved pharmacokinetics. The formation of leaky vessels and a poor lymphatic system (generally known as the EPR effect) is one of the characteristic features of cancer. Classical liposomes with a typical particle size of <200 nm provide passive drug delivery in tumorous tissue by passing through the vascular fenestrations and the impaired lymphatic drainage in the tumor to prolong the drug retention time [107,108]. Recent clinical trials revealed that this EPR effect phenomenon by itself is not as effective as expected due to the complicated structure of the tumor site (e.g., irregular vascular distribution, high tumor interstitial fluid pressure, etc.) [109]. Endosomal entrapment of long-circulating liposomes and insufficient drug release in the tumor cells are some of the factors that reduce treatment efficacy. This brought to the fore the strategy of designing functionalized liposomal carriers for specific drug targeting and site-specific drug release. Next, we focus on thermosensitive liposomes (TSL), which provide hyperthermia-triggered drug release for cancer treatment.

#### 4.4.1. Hyperthermia and Cancer

Hyperthermia can be roughly defined as a rise in tissue temperature above normal physiological levels. Inspired by the regional temperature increase that occurs naturally in various diseases, the idea of designing heat-sensitive systems has emerged [110]. Hyperthermia is known as a synergistic factor that increases the efficacy of chemotherapy and radiotherapy [111,112]. A hyperthermia technique in cancer involves external heating of the tumor via photothermal therapy, magnetic hyperthermia, or high-intensity focused ultrasounds [113]. The essential hyperthermia methods are ablation, which includes high temperature application at short intervals (>50 °C), and mild hyperthermia, which comprises the application of a lower temperature for a longer time (39–42 °C) [114]. Thermal ablation alone has the capacity to cause apoptosis and necrosis of tumor cells but significantly injures healthy non-tumorous tissues. Therefore, the use of a mild hyperthermia-drug delivery system combination gains importance at this point.

#### 4.4.2. Thermosensitive Smart Liposomes as Drug Carriers

**Figure 17:**
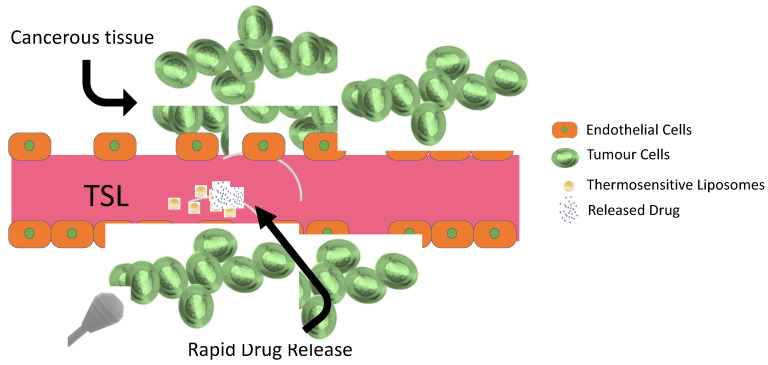
Drug release mechanism in combination with hyperthermia and thermosensitive liposomes.

Thermosensitive smart liposomes (TSL) are a class of liposomes specifically designed to release their drug content into tumorous tissue by preventing systemic drug release (Figure 17). TSL are stable at body temperature, but hyperthermia induces structural changes and disorganization in the liposomal structure [115]. Increased temperature also enhances the blood flow and vascular permeability of the tumoral cells and promotes direct cytotoxicity via TSL [116]. The concept of using TSL as drug carriers was initiated with traditional TSL in 1978, and other advanced systems were developed [117].

**Figure 18:**
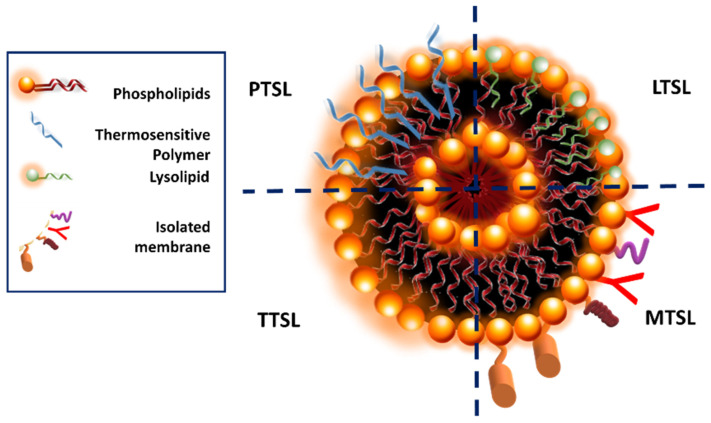
Simplified diagram of different TSL structures.

Depending on the composition, various types of TSL are obtained (Figure 18). Traditional thermosensitive liposomes (TTSL) were initially developed to provide drug release as a response to the membranal phase transition induced by temperature. The main component of liposomes is phospholipids, and various phospholipids undergoing phase transitions were used to obtain TTSL. In order to provide site-specific drug release via this first-generation TSL, phospholipids with a transition temperature (Tm) of 39–42 °C were used. TTSL triggers drug release by triggering the transition of the lipidic liposomal membrane from gel phase to liquid crystalline phase with the variation in physiological temperature. Dipalmitoyl phosphatidylcholine (DPPC)-based TTSL was prepared by adding distearoyl phosphatidylcholine (DSPC) as the co-lipid to prepare TTSL with a Tm at the desired temperature range. However, several problems encountered with TTSL, including rapid clearance by the reticuloendothelial and immune systems, a slow drug release rate, and drug leakage, revealed the need for these systems to be further improved [118].

As a strategy to improve the performance of TTSL, the incorporation of thermosensitive molecules into the liposomal membrane is studied, and in particular, polymers are used. Thermoresponsive polymers can be categorized into two groups: polymers that present a lower critical solution temperature (LCST) and polymers that present an upper critical solution temperature (UCST) [119]. Thermosensitive polymers exhibiting an LCST are used in TSL. At normal temperatures, these polymers are soluble in aqueous medium, but as temperature increases above the LCST, their solubility decreases, and the coil-to-globule-to-aggregate transition is observed [120]. This conformational transition of the polymers incorporated into the TSL structure leads to disruptions in the liposomal bilayer and triggers rapid content release.

Poly(N-isopropylacrylamide) (PNIPAAm) presents an LCST at around 32 °C, and upon its copolymerization with hydrophilic or hydrophobic monomers, it is possible to adjust its solubility and LCST. An approach is the copolymerization of N-isopropylacrylamide (NIPAAm) with hydrophobic monomers. Several researchers provided evidence that temperature triggers drug release from liposomes upon the introduction of NIPAAm-modified polymers to the liposomal structure. Moieties such as octadecyl acrylate (ODA), acrylamide (AAm), N-acryloylpyrrolidine (Apr), N,N-dimethylacrylamide (DMAM), N-isopropylmethacrylamide (NIPMAM), and propyl acrylic acid (PAA) were used for this purpose [121].

Small bioactive lipid molecules known as lysolipids are incorporated into liposomes to obtain lysolipids containing TSL (LTSL). Lysolipids are composed of a single carbon chain and a polar head group. The lysolipids tend to form highly curved micelles on the liposomal bilayer at elevated temperatures and induce drug release. Specific ligands such as folate, antibodies, and peptides can be conjugated to the surface of TSL, and therefore modified TSL (MTSL) is obtained. MTSL provides targeted drug delivery and increases the bioavailability of encapsulated agents.

Recent studies support the use of various TSL types for cancer treatment. As shown in Table 7, there is no licensed TSL formulation on the drug market at present, although some successful research results have been obtained. The probable reason for this is the complexity of TSL and the need for further research. However, it is thought that TSL will certainly help improve existing cancer treatments in the following years.

## 5. Case Study: Delivery Systems for Oral Cancer and Associated Diseases

Good oral health is of major importance for general health and well-being. The oral cavity is the first part of the digestive system and comprises various structures, including the gingiva (gum), teeth, palate, tongue, mucosal membrane, lips, and other supporting tissues [126,127,128]. The large variety of external foods and liquids going through it and its contact with air make the oral cavity a complex cavity characterized by its humidity, microbiota (bacteria, viruses, fungi, etc.), and a wide variety of tissues where several hundred species of microorganisms are located, some of which are responsible for oral health [129].

**Figure 19:**
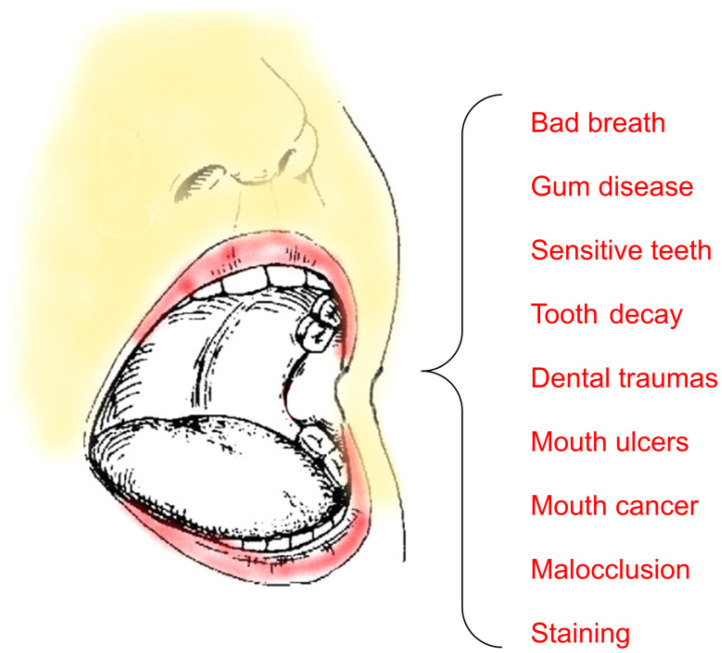
Some common oral diseases.

Alterations of the oral microbiota, microenvironment, and external factors such as systemic diseases, injuries, changes in diet, and inadequate oral hygiene may lead to various diseases, including oral cancer and oral infections (e.g., dental caries and periodontal diseases). Figure 19 shows some of the most common diseases that affect the oral cavity [129,130].

Oral cancer pertains to the cancer of the mouth itself, the oropharynx, and the lips, and in relative terms, it is the sixth most common cancer [129,131]. Effective therapeutic effects of antioxidants and other drugs can be achieved by delivering antioxidants (or other drugs) directly to the oral cavity; however, the greatest challenge for local antioxidant delivery is the short residence time in the oral cavity due to basic physiological functions such as swallowing, drinking, and/or eating. Although the most successful research on drug delivery has been achieved in the treatment of periodontitis, there has been some progress for cancer treatments that include the use of responsive liposomes and nanoparticles, which have the potential to attain drug targeting for the treatment of oral cancer [129].

In general, antioxidant therapy is closely related to the prevention of growing bacteria and the promotion of tissue regeneration. Drugs can be administered locally or systemically through delivery systems. Some of their advantages and disadvantages are displayed in Table 8. Here we will focus on the local delivery systems, as systemic drug administration is controversial in some cases because it can cause other problems; for instance, systemic antimicrobials can be the origin of antimicrobial resistance.

**Figure 20:**
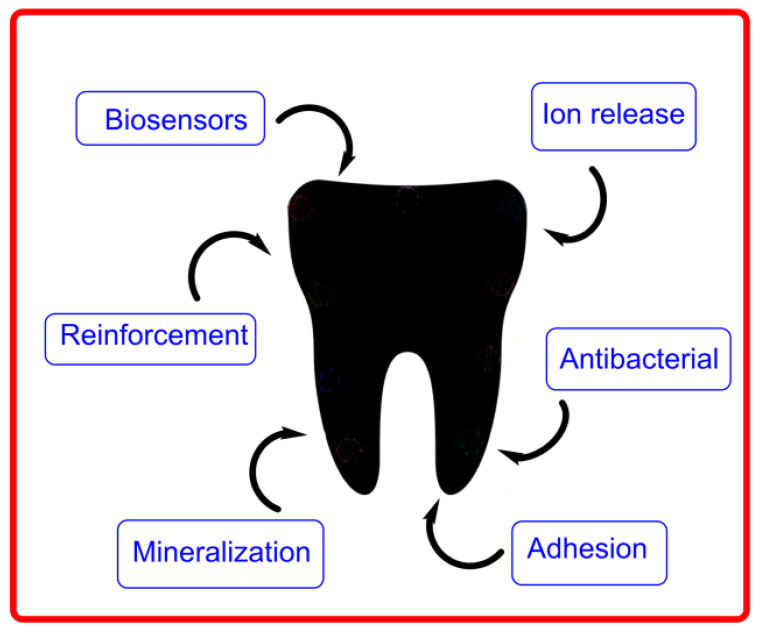
Some tooth properties that can be enhanced in dentistry with the aid of delivery systems. Adapted from V. Bonilla-Represa et al. [133].

Most oral disorders can be treated locally without the need for systemic distribution of drugs or their ingestion in the body. Local delivery of drugs into the oral cavity has several advantages because it targets areas of interest with minimum systemic adverse reactions; nevertheless, restrictions in the administration values and/or taste sensations may somewhat limit the delivery of the intended concentrations of drugs into the oral cavity. Nowadays, there is a great interest in novel delivery systems for the oral cavity to develop new therapeutic strategies (Figure 20).

Current treatment modalities for oral cancer are radiation, surgery, and chemotherapy. Radiation and surgery are effective in treating cancer in localized areas but may result in post-therapeutic complications and high morbidity in patients that may develop salivary and gland damage, osteonecrosis, mucositis, and, in general, an increase in the risk of infections. Chemotherapy is much less invasive and more efficient at treating metastatic cancer. Nonetheless, it is not risk-free, as it may induce adverse effects. Several approaches have been developed and are currently employed, including preparations in the form of tablets and other delivery systems such as liposomes and nanoparticulates. Most recently, the delivery of biopharmaceuticals such as nucleic acids, antibodies, and proteins has shown promising therapeutic results in cancer therapy, although the clinical advancement is limited [134].

Oral cancer is a superb target organ for chemoprevention because the oral cavity is easily accessible, and the advancement of the disease has a highly characteristic and recognizable presentation even from the earliest stages. In the first approach, all drug delivery systems are composed of a therapeutic agent and a transporter that carries and releases the incorporated drug. Most recently, biomaterial-based new drug delivery systems have been developed; for instance, the use of polymeric materials to promote integration of dental issues and the use of tunable materials (e.g., composite fillings employed to deliver fluoride ions, hydrogels, nanofibers, etc.) in regenerative dentistry are part of recently developed strategies to promote the formation of healthy tissues in the oral cavity.

**Figure 21:**
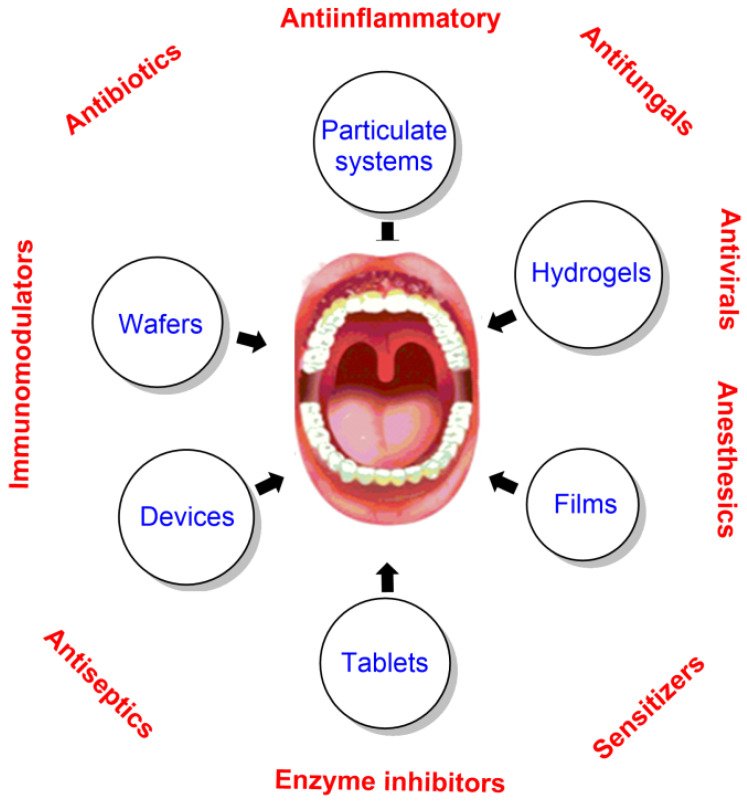
Therapeutics and some of the most common biocompatible drug delivery systems employed in dentistry. Adapted from Senel et al. [132].

Biocompatible materials, e.g., biopolymers such as gelatin, chitosan, and collagen, are widely employed in dentistry because they contact directly with biological tissues, improving body function and replacing organs and tissues. Some recent biocompatible materials employed in drug delivery systems are indicated in Figure 21.

Current strategies are based on the integrated use of natural polymeric formulations to target antibiotics and other drugs in areas of interest. Biomaterials such as chitosan and gelatin are widely employed to deliver drugs because they are absorbable drug carriers, and the surfaces of implants remain to attain convenient osseointegration. When loaded with antimicrobial substances, these biomaterials have promising effects on eradicating infections and constituting an effective treatment against a broad range of bacterial infections with little resistance to any strains. Inorganic materials such as calcium phosphate are widely employed in maxillofacial surgery and dentistry. These inorganic materials are usually composed of calcium pyrophosphates (P_2_O_7_^4−^), metaphosphates (PO_3_^−^), and orthophosphates (PO_4_^3−^). Pyrophosphates are chelating agents with low toxicity and a broad range of applications. Like other chelating agents, their prime function is to combine with metal elements and, in toothpastes, inhibit calcium phosphate deposits in the form of dental calculus [135]. When loaded with antibiotics, these provide a useful tool to prevent infectious situations because the kinetics of antimicrobial release can be controlled by both the surface and the porosity of the matrix. The functional groups of antibiotics (-NH_2_, -OH, -COOH, etc.) are responsible for the interactions with the Ca^2+^ and PO_4_^3−^ in the matrix, so that the mechanism and kinetics of release are affected by pH, temperature of degradation, chemical structure, and solubility of the drug.

Metallic nanoparticles can be combined with polymers or coated with biomaterial surfaces. These particles reveal superior antimicrobial properties in the oral cavity [132,134,135]. The high surface-to-volume ratio of nanoparticles has also been exploited in the medical sciences, especially in dentistry and dental surgeries. Titanium, gold, and silver nanoparticles have been employed as nanocarriers because of their physical, optical, and mechanical properties. Titanium-based nanoparticles are usually employed in orthopedic or dental bone implant osseointegration, particularly those with TiO_2_, which stimulate adhesion of cells, wound healing, and migration of cells. TiO_2_ nanoparticles improve the loading of efficient anti-inflammatory drugs such as ibuprofen and gentamicin. Gold-based nanoparticles have also been widely employed in dentistry, mainly in the fields of periodontology, restorative dentistry, dental implants, and cancer diagnosis. Silver nanoparticles are commonly used for their antibacterial properties. Silver nanoparticles show inhibitory effects that are believed to be related to bacterial cell disruption, inhibition of DNA synthesis, and permeability. Their effects are dosage-dependent. The complexation of silver nanoparticles with polyethylene imines and metronidazole enhances the antibacterial and antimicrobial effects of the nanoparticles. Currently, zirconia nanoparticles are being explored as an alternative to the titanium ones because they show similar biocompatibility and mechanical advantages. Metallic nanoparticles prepared with other metals such as bismuth (e.g., bismuth subsalicylate) also inhibit the growth of several periodontal pathogens, including *A. actinomycetemcomitans*, *C. gingivalis*, and *P. gingivalis* [136].

In implant-mediated drug delivery systems, the drug is released and received in the targeted tissue without any pain. The intramuscular, subcutaneous, and intravenous routes of administration have several advantages over the gastrointestinal route, despite the fact that the latter is probably the most widely used route because of its practical and economic benefits. Alternatively, the various parenteral administration routes allow the rapid activity of drugs with high bioavailability and moderate economic cost, permitting continuous usage of intravenous injections [4,6].

Emulsions, nanoemulsions, vesicles, nanovesicles, and liposomes are largely used as delivery systems of a variety of therapeutic agents, anticancer drugs, bioactives, and antimicrobials because they can be easily prepared by employing biocompatible components and are cost-effective. Emulsions and nanoemulsions constitute a group of colloidal organic nanosized systems composed of two immiscible liquids (emulsions and nanoemulsions), one dispersed in the other, that need the input of mechanical energy to be formed. Nanovesicles and liposomes are spherical colloidal systems containing an aqueous core surrounded by a bilayer membrane that are formed spontaneously by the self-assembly of surface-active molecules such as phospholipids, nonionic surfactants, and amphiphilic block copolymers. Liposomes were first prepared with phospholipids and cholesterol and significantly reduced their toxicity while, at the same time, improving the therapeutic action of entrapped drugs. PEGylated liposomes are currently designed by coating the liposome with polyethylene glycol and have been shown to increase the circulation time and stability of the drug, maximizing their bioavailability [136,137].

Nanoemulsions are widely employed in the prevention and treatment of diseases of the oral cavity [76]. Because of their reduced size, they can deliver drugs into deeper layers of the oral mucosa, resulting in a much better and complete cure of the disease. Nanoemulsions with a high surfactant content have also been shown to exert antimicrobial activity. For example, oil-in-water nanoemulsions prepared by mixing soybean oil, cetylpyridiniumchloride, and Triton X-100 have antimicrobial activity against cariogenic Streptococcus mutans, suggesting that this formulation can be employed for the prevention of dental caries [77].

Pickering emulsions are formed when conventional emulsions are recovered with solid particles that surround the oil core. The main difference with the conventional ones is that the Pickering emulsions are surfactant-free, but their unique structure makes them display excellent physical stability and superior biocompatibility. They show great potential in oral drug delivery, providing several-fold increases in the oral bioavailability or bioaccessibility of poorly soluble drugs such as curcumin, puerarin, and rutin. Pickering emulsions can simultaneously solubilize poorly soluble drugs and enhance their permeation across biomembranes [8], displaying great resilience to coalescence and Ostwald ripening [9,10].

## 6. Conclusions and Final Remarks

Among all the methods available to enhance the aqueous solubility of antioxidants, drug delivery systems have gained significant interest among researchers, as reflected by the number of research papers published. Formulations considered classic have a number of limitations: (1) a high amount of the drugs administered but poor bioavailability; (2) a risk of adverse effects; (3) instability during storage; and (4) a marked plasma fluctuation of the antioxidant concentration, which hinders a prolonged effect. Thus, there is a need to find a type of system that allows the release of active antioxidants in a way that is more effective and avoids side effects.

The use of drug delivery systems has emerged to enhance the therapeutic action and bioavailability of the active antioxidants at the target site, decrease their toxic side effects, and broaden their application. Solubilization and delivery of active drugs employing aqueous-based and biocompatible systems are the preferred strategies to reduce dose and frequency of administration and enhance therapeutic action. The objective in developing controlled drug delivery systems is to decrease as much as possible the dosing frequency, the amount of dose, and the targeting of the drug to the desired site of action to eventually provide uniform drug delivery.

An ideal drug delivery system should, therefore, direct drugs to the site of action, preferably with a single dose for the entire treatment period. This imaginary delivery system is, however, impossible because fluctuations in plasma concentration alter any or all of the desired properties of the delivery system.

To find adequate drug delivery systems, some considerations must be taken into account, such as the biomaterial properties (biocompatibility, hydrophobicity, degradation, and rheological properties), pharmacokinetics, stability, route of administration, drug binding efficiency with plasma proteins, the ability of the drug to cross biological barriers, and regulatory aspects, among others.

This review focuses on the structure, characteristics, and applications of different drug release systems. The benefits and mechanisms of controlled drug release are explained, and as a case study, drug delivery to the oral cavity was examined in detail. Drug delivery systems are proven to improve the pharmacological performance of therapeutic agents. The unique properties of these systems, including controlled drug release, prolonged plasma half-life, and targetability, have encouraged the researchers to focus on this topic. Consequently, their industrial manufacture and medical implementation are growing. With the recent developments in polymer technology, nanotechnology, and biotechnology, it is thought that superior drug delivery strategies will be developed to treat a wide range of diseases.

Considerable advances in drug delivery have occurred within the past few years. Extended release, controlled release, and other formulations are now available for a large variety of drugs. A recent example of a delivery system are the recently developed COVID-19 vaccines designed to provide immunity against the severe acute respiratory syndrome (SARS-COVID-19). Related to vaccines, global vaccine programs are becoming a reality with the use of oral, transmucosal, transcutaneous, and needle-less vaccination. New drug delivery systems will develop in the next decade through the desirable and necessary interdisciplinary collaboration of researchers. Future challenges will include economic factors, a detailed characterization of physiological barriers (e.g., the blood-brain barrier), and, obviously, the development and implementation of new drugs with a broad action spectrum in new delivery systems. Progress in the development of preclinical antioxidant evaluation should provide an understanding of its stability, efficiency, and safety upon administration, leading to a more successful clinical translation of these and other drug delivery systems and thereby developing new possibilities addressed towards a personalized medicine.

## Figures and Tables

**Table 1:** Redox potential of ROS and some ROS scavengers (listed from highly oxidizing to highly reducing characteristics) relative to the standard hydrogen electrode. RS: cysteine, GSH: glutathione, and PUFA: polyunsaturated fatty acid [25,27,28].

Radical Couple	*E*_p_^0^ (mV)
**^∙^** **OH, H^+/^H_2_O**	2330
**O_3_** **^∙^** **^−^** **, 2H^+^/H_2_O + O_2_**	1800
**RO** **^∙^** **, H^+^/ROH**	1600
**HOO** **^∙^** **, H^+^/H_2_O_2_**	1060
**ROO** **^∙^** **, H^+^/ROOH**	1000
**O_2_** **^∙^** **^−^** **, H^+^/H_2_O_2_**	940
**RS** **^∙^** **/RS^-^**	920
**GSH/GS** **^∙^**	920
**O_3/_O_3_** **^∙^** **^−^**	890
**^1^** **O_2/_^1^O_2_** **^∙^** **^−^**	650
**PUFA** **^∙^** **, H^+^/PUFA**	600
**Catechol-O** **^∙^** **/catechol-OH**	530
**α** **-Tocopheroxyl** **^∙^** **, H^+/^** **α** **-Tocopherol**	500
**Trolox** **^∙^** **, H^+^/Trolox-OH**	480
**H_2_O_2_, H^+^/H_2_O, HO** **^∙^**	380
**Ascorbate^−^** **^∙^** **, H^+^/Ascorbate**	282
**O_2/_O_2_** **^∙^** **^−^**	−160
**H_2_O/e^−^ (aq)**	−2870

**Table 2:** Physicochemical properties of some cyclodextrins (CDs). *P*_W_^OCT^ is the partition constant of CD between the octanol and water phases [15].

	External Diameter (Å)	Internal Diameter (Å)	Solubility (mg/mL), T = 25 °C	log (*P*_W_^OCT^)	Surface Tension (mM/m)
**α-CD**	14.6	4.7–5.3	145	−13	71
**β-CD**	15.4	6.0–6.5	18.5	−14	71
**Randomly methylated β-CD**	---	---	>500	---	62
**Dimethyl-β-CD**	---	---	570	−6	57.5–54.1
**2-Hydroxypropyl-β-CD** **(HP--β-CD)**	15.4	6.0–6.5	>1200	−11	54.8–57.5
**Sulfobutylether-β-CD sodium salt (SBE--β-CD)**	---	---	>1200	−10	71
**δ-CD**	17.5	7.5–8.3	232	−17	71
**2-Hydroxypropyl-δ-CD**	---	---	800	−13	71

**Table 3:** Some cyclodextrin-based delivery systems for cancer therapy that have been investigated.

CD-based Nanocarrier	AO with Reported Anticancer Properties	Type(s) of Cancer	Reference
**Sulfobutylether-β-CD**	Resveratrol	Lung	[45]
**Sulfobutylether-β-CD**	Celastrol	Lung	[46]
**α-, β-, and γ-CD**	New Zealand propolis	Esophageal squamous cell, colon, gastric, and colorectal adenocarcinomas	[47]
**D-α-tocopherol polyethylene glycol 1000 succinate-modified β-CD**	Genistein	Breast	[48]
**β-CD**	Carvacrol	Prostate	[49]
**α-, β-, and γ-CD**	Curcumin	Lung, prostate, breast, and colorectal	[50]

**Table 4:** Advantages and limitations of controlled drug delivery systems.

Advantages	Limitations
Provides a uniform therapeutic response by stabilizing the blood drug concentration and reducing the fluctuations in plasma concentration; Improves patient compliance. The patient care period in hospitals can be reduced; As the drug intake frequency per day/month decreases, the number of missed doses also decreases; Reduces local and systemic toxicity by providing localized drug release and reducing the total drug intake, thus providing maximum bioavailability with the minimum dose; The physicochemical stability of the active molecules can be provided (e.g., protection against enzymatic inactivation or bacterial decomposition via encapsulation); Drugs with a short plasma half-life can be administered at longer dosage intervals; Advantageous for the manufacturer in terms of increased commercial value.	Immediate termination of the therapy just after drug administration is not always possible; The dosage is adjusted by considering the average pharmacokinetic behavior in the normal population; therefore, the dose regimen cannot be freely modified; The sterility necessity of the implants and their application/removal by surgical operation are disadvantages in terms of manufacturing and ease of use; It is not possible to design a controlled-release dosage form for each active molecule; its physicochemical and biological properties must be considered; Research and development costs are higher in terms of manufacturing and equipment.

**Table 5:** Most commonly used biodegradable and non-biodegradable polymers in CDDS.

Biodegradable Polymers	Aliphatic polyesters: poly(glycolic acid), poly(lactic acid), poly (lactic-co-glycolic acid), poly-ε-caprocalctone, poly(p-dioxanone), and poly(orthoesters); Poly(amino acids): poly(L-lysine) and poly(glutamic acid); Polyphosphazenes: aryloxyphosphazenes; Biodegradable polyurethanes; Polyanhydrides: poly(sebacic acid-co-1,3-bis(p-carboxyphenoxy) propane); Peptides and proteins: collogen, gelatin, elastin, keratin, silk, and proteoglycans; Polysaccharides: cellulose, starch, alginate, gellan gum, glycosaminoglycans, chitosan, and inulin; Polyhydroxyalkanotes: poly(3-hydroxyburtyrate); Lignin.
Non- Biodegradable Polymers	Poly (ethylene-co-vinyl acetate); Polysiloxanes (silicones); Non-biodegradable polyurethanes; Polythene.

**Table 6:** Several examples of marketed CDDS for cancer therapy.

Product Name	Active Ingredient	Indication
Lupron Depot	Leuprolide	Treatment of prostate cancer and endometriosis
Sandostatin LAR	Octreotide	Treatment of acromegaly and endocrine tumors
Trelstar Depot	Triptorelin pamoate	Palliative treatment of advanced prostate cancer
Zoladex	Goserelin acetate	Advanced prostate cancer and advanced
Gliadel	Carmustine	Brain tumors
Decapeptyl	Triptorelin acetate	Prostate cancer
Profact Depot	Buserelin acetate	Prostate cancer

**Table 7:** Recent TSL formulations evaluated for cancer therapy.

TSL Type	Cargo	Preparation Method	TSL Composition *	Study Outcome	Ref.
LTSL	Mistletoe lectin-1 (ML1)	Film hydration and extrusion.	DPPC:MSPC:DSPE-PEG2000 (86:10:4%mol).	The bioactivity of TSL against murine CT26 colon carcinoma cells in terms of cytotoxicity and inhibition of tumor cell viability was shown.	[122]
TTSL LTSL	Gadolinium-DTPA-BMA)	Film hydration.	DPPC:MSPC:DSPE-PEG2000 (85:10:5).	Gd-DTPA-BMA-loaded TSL exhibited improved cytotoxicity against breast cancer cell lines compared to free Gd-DTPA-BMA, and the low cytotoxicity of TSL on normal cell lines (WI-26 VA 4) revealed the selectivity of the carrier.	[123]
LTSL	Hypericin	Film hydration.	DPPC:DSPC:DSPE-mPEG-2000: Hypericin (79:15:5:1).	Safety of hypericin-loaded TSL was shown by hemocompatibility studies; the combination of heat and TSL led to increased ROS levels, enhanced intracellular uptake, and phototoxicity in breast cancer originated from MDA-MB-231 cells.	[124]
MTSL	siRNA	Film hydration and extrusion.	DPPC:MSPC:DSPE-PEG2000 (165:5.5:5) macrophage membrane DSPE-PEG2000-cRGD CPP.	Macrophage membrane- and cRGD-functionalized thermosensitive liposomes combined with CPP provided tumor-targeted delivery of siRNA in tumor-bearing mice and tumor inhibition efficacy was shown both in vitro and in vivo.	[125]

* DPPC: 1,2-dipalmitoyl-sn-glycero-3-phosphocholine; DSPE-PEG2000: 1,2-distearoyl-sn-glycero-3-phosphoethanolamine-NPEG2000; MSPC: 1-stearoyl-2-hydroxy-sn-glycero-3-phosphocholine; DSPC: 1,2-distearoyl-sn-glycero-3-phosphocholine; cRGD:cyclic Arg-Gly-Asp peptide; and CPP: cell-penetrating peptide.

**Table 8:** Some local and systemic drug delivery systems for the oral cavity [130,132].

	Advantages	Disadvantages
Local drug delivery system	- Minimum side effects; - Minimum adverse reactions; - Delivery at a specific area; - Prolonged time of contact (reduction of dose and times of administration).	- Restriction of the route of administration value; - Taste disturbances; - Restricted surface area; - Poor tissue penetration; - Rapid removal.
Systemic drug delivery system	- Broad distribution.	- Systemic undesirable reactions; - Antimicrobial resistance; - Dysbacteriosis; - Limited delivery in oral lesions.

## Data Availability

Not applicable.
